# Fracture Behavior of Short Fiber-Reinforced Direct Restorations in Large MOD Cavities

**DOI:** 10.3390/polym13132040

**Published:** 2021-06-23

**Authors:** Márk Fráter, Tekla Sáry, Eszter Vincze-Bandi, András Volom, Gábor Braunitzer, Balázs Szabó P., Sufyan Garoushi, András Forster

**Affiliations:** 1 Department of Operative and Esthetic Dentistry, Faculty of Dentistry, University of Szeged, H-6720 Szeged, Hungary; teklasary@gmail.com (T.S.); vbeszter96@gmail.com (E.V.-B.); 2 Volom Dental, H-1037 Budapest, Hungary; drvolom@drvolomdental.hu; 3 dicomLAB Dental Ltd., H-6726 Szeged, Hungary; braunitzergabor@gmail.com; 4 Department of Food Engineering, Faculty of Engineering, University of Szeged, H-6725 Szeged, Hungary; szpb@mk.u-szeged.hu; 5 Department of Biomaterials Science and Turku Clinical Biomaterials Center-TCBC, Institute of Dentistry, University of Turku, FI-20520 Turku, Finland; sufgar@utu.fi; 6 Urban Regeneration Institute, H-1025 Budapest, Hungary; andras.forster@me.com

**Keywords:** deep MOD cavity, flowable composite, short fiber composite, fatigue resistance

## Abstract

The aim of this research was to study the impact of using a short fiber-reinforced composite (SFRC) core on the fatigue performance and fracture behavior of direct large posterior composite restorations. Moreover, the influence of the consistency (flowable or packable) of occlusal composite coverage was assessed. A total of 100 intact molars were collected and randomly distributed into five groups (n = 20). Deep mesio-occlusal-distal (MOD) cavities were prepared in all groups. After adhesive treatment and rebuilding the missing interproximal walls with conventional composite, the specimens in four experimental groups were restored by an SFRC core (everX Flow), which was applied and cured either in bulk or in oblique layers (each 2 mm thick). Packable (G-aenial Posterior) or flowable (G-aenial Injectable) conventional composites were used as a final occlusal layer. The control group was restored with only packable conventional composite. Fatigue survival was measured for all specimens using a cyclic loading machine until a fracture occurred or a total of 25,000 cycles was achieved. Kaplan–Meyer survival analyses were conducted, followed by pairwise log-rank post hoc comparisons. The static load-bearing capacity of surviving teeth was tested using a universal testing machine. Fracture patterns were evaluated visually. There was no statistically significant (*p* > 0.05) difference in terms of survival between the tested groups. All groups for which flowable SFRC was used showed statistically significantly higher load-bearing capacities compared to the control group (*p* < 0.05). There were no significant differences regarding fracture resistance among the fiber-reinforced study groups. Regarding the fracture pattern during the survival analysis, all specimens that received SFRC showed a dominantly restorable type of fracture, while the control specimens presented a dominantly non-restorable type. The use of flowable SFRC as a reinforcing core for large MOD direct restorations showed promising achievements regarding fracture behavior.

## 1. Introduction

With the development of adhesive techniques and composite resin restorative materials, composite fillings have come to be routinely used in the posterior region of the mouth [1,2]. The number of direct posterior composite restorations is expected to increase further with the worldwide phasing out of the use of amalgam [3,4,5]. In addition to aesthetic considerations, modern direct restorations must also restore function and protect the remaining tooth structure against fractures [6]. The more dentin is missing from posterior teeth (due to caries or trauma), the more challenging it becomes to properly reinforce the complex formed by the remaining tooth material and the restoration. Of the possible cavities in posterior vital teeth, MOD (mesio-occlusal-distal) cavities are the most demanding. This is due to the loss of both marginal ridges, which results in a considerable loss of stiffness [7,8]. The literature suggests that standardized MOD cavity preparation in upper premolars leads to a 63% mean loss in relative cuspal stiffness [9], while the loss of only one marginal ridge results in a mean loss of only 46% in the same parameter [10].

Besides the presence or absence of the marginal ridges, the volume factor, which refers to the size (mainly the depth) of the cavity, is the other important weakening factor in the posterior cavities of vital teeth. Forster et al. managed to show that, while shallow MOD cavities could safely be restored with a direct composite filling, deep MOD cavities with a composite filling showed significantly lower fracture resistance, regardless of the thickness of the remaining walls [11]. The two main inherent problems of composite resin filling materials are polymerization shrinkage and related stress, as well as the inadequate fracture toughness as compared to the dentin [12]. Fracture toughness describes the damage tolerance of the material and can be considered as a measure of fatigue resistance, which predicts structural performance [13]. The problem of the lack of toughness is especially well seen in extensive direct restorations (e.g., deep MOD cavities), as the volume of the material used for restoration increases in these cases [14]. 

The use of state-of-the-art short fiber-reinforced composite (SFRC) materials seems to offer a solution to this problem. Sáry et al. demonstrated that deep MOD cavities can be reinforced by the application of SFRC in direct composite restorations [15]. Such restorations were also characterized by a predominantly favorable fracture pattern [15]. In 2019, the flowable version of SFRC was introduced with the promise of easy adaptability in both large cavities and limited spaces (such as root canals). So far, flowable SFRC has yielded remarkably favorable results when applied either alone in the root canal (Bioblock technique) [16] or as a luting material for post luting [17]. 

To the best of our knowledge, no one has yet tested the flowable SFRC in deep cavities in vital teeth for direct restorative purposes. The question arises as to whether the application of the material (in bulk or by layering) influences the fatigue resistance and fracture behavior of the restoration in this situation. The purpose of this laboratory investigation is to evaluate the reinforcing effect of a flowable SFRC material placed with different application methods in class II deep MOD cavities. In addition, the impact of the consistency (flowable or packable) of occlusal conventional composite coverage is assessed.

The null hypothesis is that (1) the teeth restored with the flowable SFRC would show similar mechanical resistance to teeth restored with conventional composite filling, and that (2) the fracture patterns in molar teeth with deep class II cavities would not depend on the restorative technique applied. The sum of the residual coronal structure and internal root structure defines the needs of post-usage.

## 2. Materials and Methods

The study was approved by the Ethics Committee of the University of Szeged, and the study design conformed to the Declaration of Helsinki in all respects. A total of 100 mandibular 3rd molars extracted for periodontal or orthodontic reasons were selected for this investigation. The teeth were placed in 5.25% NaOCl for 5 min immediately upon extraction and then stored in a 0.9% saline solution at room temperature until use. All specimens were used within 2 months of extraction. The soft tissue covering the root surface was removed with hand scalers before use. The inclusion criteria were a visual absence of caries or root cracks and an absence of previous endodontic treatment, posts, crowns, or resorptions. Approximately 80% of the specimens fell within the 11–11.5 mm size range (measured at the widest bucco-lingual dimension), and the rest were between 10 and 12 mm. Regarding the mesio-distal dimension, the deviation limit was ±10% from the group mean. The height of the specimens was between 8.0 and 9.0 mm, as measured from the cementoenamel junction (CEJ). The teeth were evenly divided into 5 groups (n = 20).

### 2.1. Cavity Preparation and Restorative Procedures

**Figure 1:**
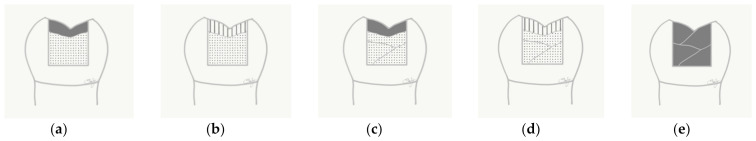
Schematic figure representing the test groups (from left to right). (**a**) Group 1: flowable SFRC bulk and conventional packable composite; (**b**) Group 2: flowable SFRC bulk and conventional flowable composite; (**c**) Group 3: flowable SFRC layered and conventional packable composite; (**d**) Group 4: flowable SFRC layered and conventional flowable composite; (**e**) Control Group: conventional packable layered composite.

All teeth received standardized MOD cavity preparation with a depth of 4.5–5 mm and a 2.5 mm wall thickness on both vestibular and oral aspects by the same trained operator, as previously described by Forster et al. [11]. The cavity was rinsed with water and air-dried with an air/water syringe. Then, a Tofflemire (1101C 0.035, KerrHawe, Bioggio, Switzerland) matrix was applied and the enamel was acid-etched selectively with 37% phosphoric acid for 15 s, followed by rinsing with water and air-drying. For the adhesive treatment of the cavity, G-Premio Bond (GC Europe, Leuven, Belgium) was used, as per the manufacturer’s instructions. The adhesive was light-cured for 40 s with a D-light Pro photopolymerization unit (GC Europe) in “HP” mode (light intensity: 1000 +/− 60 mWcm^2^). An approximately 0.5 mm-thin flow composite layer (G-aenial Flo A2, GC Europe) was applied on all walls of the cavity in all groups. This layer was light-cured for 40 s. After applying this flowable layer, highly filled low-viscosity flowable composite (G-aenial Injectable Flow A2, GC Europe) was injected into the approximal cavity margins, and packable composite resin (G-aenial Posterior A2, GC Europe, Leuven, Belgium) was placed and packed to the approximal wall of the matrix in one increment, transforming it into a class I according to the centripetal technique. This dual layer was light-cured for 40 s. The cavities were restored as follows (see Figure 1).

Group 1: The cavity was restored with a bulk injection of flowable SFRC (everX Flow Bulk, GC Europe), leaving 2 mm of space for the occlusal layer. The occlusal aspect was restored cusp by cusp in 2 mm-thick oblique increments of packable composite resin (G-aenial Posterior A2, GC Europe). Each increment was light-cured from the occlusal surface for 40 s and, after the removal of the Tofflemire matrix, the mesial and distal sides were also light-cured for 20 s each (total curing time: 80 s). 

Group 2: The central part of the cavity was restored in the same way as described in Group 1. The occlusal aspect was restored cusp by cusp in 2 mm-thick oblique increments of highly filled low-viscosity flowable composite (G-aenial Universal Injectable Flow A2, GC Europe). The light curing of each increment and the mesial and distal sides after removing the matrix was performed in the same way as in Group 1.

Group 3: The central part of the cavity was restored with oblique increments (each maximum 2 mm thick) of SFRC Flowable (EverX Flow Bulk, GC Europe), leaving 2 mm of space for the occlusal layer. The occlusal aspect was restored cusp by cusp in 2 mm-thick oblique increments of packable composite resin (G-aenial Posterior A2, GC Europe). The light curing of each increment and the mesial and distal sides after removing the matrix was performed in the same way as in Group 1.

Group 4. The central part of the cavity was restored in the same way as described in Group 3. The occlusal aspect was restored cusp by cusp in 2 mm-thick oblique increments of highly filled low-viscosity flowable composite (G-aenial Universal Injectable Flow A2, GC Europe). The light curing of each increment and the mesial and distal sides after removing the matrix was performed in the same way as in Group 1.

Control Group: The central part of the cavity was restored with consecutive 2 mm-thick oblique increments of packable composite resin (G-aenial Posterior A2, GC Europe). The light curing of each increment and the mesial and distal sides after removing the matrix was performed in the same way as in Group 1.

The restorations were finished with a fine granular diamond burr (FG 7406-018, Jet Diamonds, Ft. Worth, TX, USA and FG 249-F012, Horico, Berlin, Germany) and aluminum oxide polishers (OneGloss PS Midi, Shofu Dental GmbH, Ratingen, Germany). The restored specimens were stored in physiological saline solution (Isotonic Saline Solution 0.9% B.Braun, Melsungen, Germany) in an incubator (mco-18aic, Sanyo, Japan) at 37 °C until the start of the experimental procedures.

### 2.2. Mechanical Testing

Prior to embedding, the root surface of each tooth was coated with a layer of liquid latex separating material (Rubber-Sep, Kerr, Orange, CA, USA) to simulate the periodontal ligament. The specimens were then embedded in methacrylate resin (Technovit 4004, Heraeus Kulzer, Hanau, Germany) 2 mm from the CEJ. This was performed to simulate the bone level. Mechanical testing was carried out in two phases. In the first phase, all restored specimens were submitted to an accelerated fatigue-testing protocol [16,17] by a hydraulic testing machine (Instron ElektroPlus E3000, Norwood, MA, USA) placed parallel to the long axis of the tooth. This phase served the purpose of simulating normal biting forces. Cyclic isometric loading was applied with a round-shaped metallic tip (6 mm in diameter) to the center of the occlusal surface of the crown in the central pit, between the buccal and oral cusps. A cyclic load was applied at a frequency of 5 Hz, starting with gradually increasing the static loading till 200 N in 5 s, followed by cyclic loading in 200 N steps up to 1000 N, with 5000 cycles per step. The specimens were loaded until fracture occurred or up to 25,000 cycles. The total number of cycles survived was recorded for each specimen for the survival analyses. In the second phase, the surviving specimens underwent static load-to-fracture testing (Lloyd R1000, Lloyd Instruments Ltd., Fareham, UK) at a cross-head speed of 2 mm/min. In this phase, traumatic forces were simulated. A force vs. extension curve was dynamically plotted for each tooth. Fracture threshold—defined as the load at which the tooth–restoration complex exhibited the first fracture, resulting in a peak formation on the extension curve—was recorded in Newtons (N). 

Finally, each specimen was visually examined to determine the type and location of failure, as well as the direction of the failure. Fractures were classified according to Scotti and co-workers, based on optical microscopic examination, with a two-examiner agreement. A restorable fracture was defined as one above the CEJ, while a non-restorable fracture was defined as one extending below the CEJ [18].

### 2.3. Statistical Analysis

Statistical analysis was performed in SPSS 23.0 (IBM Corp., Somers, NY, USA). The number of cycles survived was analyzed descriptively for each group and with the Kaplan–Meier method across the groups (with the Breslow test for the pairwise analyses). The frequency of restorable and non-restorable fractures as well as the number of surviving teeth were calculated for each group. For the comparisons between the surviving samples, ANOVA with Tukey’s HSD post hoc test was used. The general limit of significance was set at α = 0.05. 

## 3. Results

**Figure 2:**
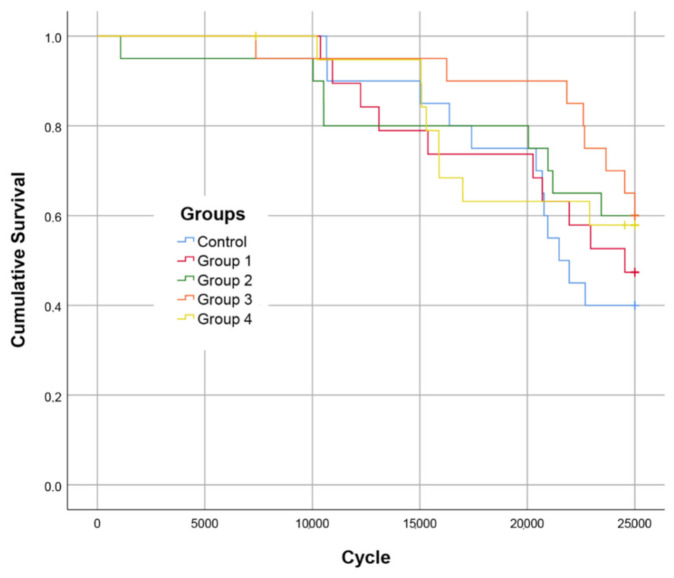
Fatigue resistance survival curves (Kaplan–Meier survival estimator) for all tested groups.

The Kaplan–Meier survival curves are presented in Figure 2. Table 1 displays the *p* values for group-wise comparisons regarding the cyclic loading test. 

There was no statistically significant difference in terms of survival between the tested groups. Regarding the fracture pattern, all specimens with restorations utilizing flowable SFRC showed a dominantly restorable type of fracture, while the control group presented dominantly non-restorable ones (Table 2).

**Figure 3:**
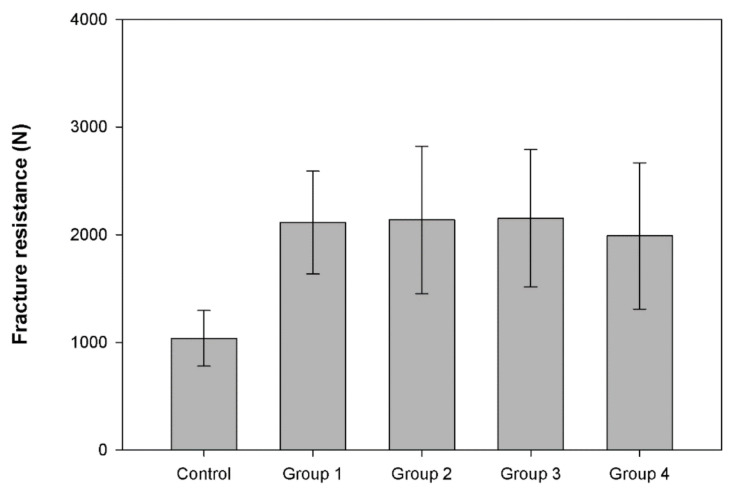
Fracture resistance mean values (N) and standard deviations of surviving test restorations.

Figure 3 shows the average fracture resistance values of the previously surviving specimens under static loading. All groups using flowable SFRC showed statistically significantly higher fracture resistances compared to the control group. There was no significant difference regarding fracture resistance among the fiber-reinforced study groups.

## 4. Discussion

This in vitro investigation aimed to compare the possible reinforcing effect of flowable SFRC applied either in bulk or in a layered manner compared to conventional composite fillings in deep, endodontically non-treated MOD cavities. It was also assessed whether the consistency of the composite used for covering the SFRC material is important in terms of fatigue failure and fracture resistance. First, cyclic loading was applied in the form of an accelerated fatigue test to all specimens. It is known that cycling fatigue loading simulates the clinical situation better than static loading, as it generates cyclic forces similar to normal masticatory forces. This protocol (accelerated fatigue) was introduced as a rational middle ground between the classic load-to-fracture test and the more sophisticated and time-consuming fatigue tests [19,20,21].

In the posterior region, forces range from 8 to 880 N during normal mastication [22]; thus, the accelerated fatigue test was only performed up to 1000 N. Some studies have used higher loads with this test [21,23], but in this specific situation it would have been unrealistic for the said reason. We thus consider the applied method to be a definite strength of this study.

Regarding survival, while the control group clearly showed the lowest survival rates, there were no statistically significant differences among the tested groups. To the best of our knowledge, direct restorations utilizing flowable SFRC in teeth have not been tested by cyclic loading so far. The results suggest that neither the use of flowable SFRC nor the consistency (flowable or packable) of the occlusal conventional composite coverage could significantly improve the fatigue resistance of direct MOD restorations compared to conventional composite fillings. However, SFRC seems to shift the fracture pattern toward predominantly restorable. This latter finding is in line with the results of other studies in that it shows that the use of SFRC, should fracture occur, allows a more favorable fracture profile than composite without fiber reinforcement [15,24,25,26,27]. This is due to the obvious difference in fracture toughness between reinforced and non-reinforced composites. Previous studies have shown that fiber-reinforced composites have the ability to re-direct and stop crack propagation within the materials [13,17,27]. 

**Figure 4:**
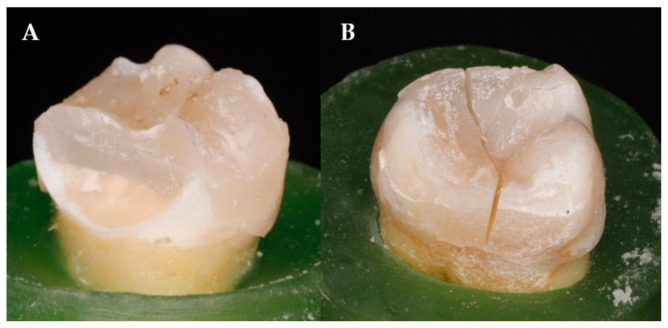
Examples of failed specimens. Picture (**A**) shows a favorable repairable fracture (in the case of SFRC), while picture (**B**) shows an unfavorable, irreparable fracture going through the direct restoration (lack of SFRC).

In fact, the presence of such energy-absorbing and stress-distributing fibers allows crack propagation to be deflected away from the bulk of the material and toward the peripheries (Figure 4A). On the other side, the brittleness of the conventional composites generated the bulk fracture which propagated easily through the whole thickness of the restoration (Figure 4B). Thus, the basic characteristics of the material do not significantly enhance the resistance of fatigue crack propagation.

Upon the completion of the accelerated fatigue test, a load-to-fracture test was performed on the surviving specimens. The load-to-fracture test, given the high applied load, is similar to modelling traumatic injury to the restoration–tooth complex (e.g., biting accidentally on a seed, stone, etc.). In the static load-to-fracture test, all bi-structured restorations (SFRC + conventional composite coverage), irrespective of the application mode of flowable SFRC or the consistency of the occlusal composite material, showed significantly higher fracture toughness in comparison to the control group (conventional composite filling). This is in accordance with the findings of Garoushi et al., where flowable SFRC covered with a minimal amount of composite showed significantly higher fracture resistance compared to conventional composite filling [28]. However, in that study the cavities were larger than those seen in this study. Furthermore, the current findings contradict the findings of Sáry et al. [15] and also our previous findings [24], where there was no statistically significant difference in terms of fracture resistance between an MOD cavity bulk-filled with SFRC compared to a layered composite filling. 

It must be mentioned, though, that in neither of the mentioned studies was the same flowable SFRC used as in this one. This contrast could be due to the unique structure and high fiber content of the flowable SFRC material. While packable SFRC utilizes millimeter-long fibers, those in the flowable SFRC are micrometer-long. Even with the smaller fibers, their aspect ratio, which refers to the length compared to the diameter of the fiber (l/d), is within the range of 30 [13]. Therefore, it holds the promise of reinforcement to the materials and also to the adhered dental tissues. Another major difference between the packable and flowable SFRC is that the flowable one contains 25 wt% of fibers, while in the packable variant this ratio is only 9 wt%. So far, flowable SFRC has yielded promising results when utilized in direct restorations in different clinical situations [16,17,28].

Our results show the superiority of bi-structured direct restorations over conventional composite fillings when tested with extremely high forces. This is in line with other studies [26,28]. Our results also suggest that the consistency (highly filled flowable or conventional packable) of the occlusally placed composite is not a significant factor in the fracture resistance of a direct restoration utilizing flowable SFRC. This could be due to the improved mechanical properties of the flowable composite resin we used. In fact, both conventional composite resins have similar fracture toughness values of 1.1 MPam^1/2^, which is much lower than the values of SFRC 2.6 MPam^1/2^ [27]. 

G-aenial Universal Injectable (GC Europe) contains 69 wt% filler, making it suitable for direct restorations without any further coverage. Our results are in line with the clinical findings of Lawson et al., but it must be mentioned that they did not use bi-structured restorations for their study [29].

A known limitation of our study is that only one specific material’s application (i.e., flowable SFRC) was investigated and compared with the most frequently used type of direct restoration—namely, direct composite filling. In future, other composite materials should be addressed in the same study setup.

## 5. Conclusions

According to our results, deep MOD cavities can be restored with both fiber-reinforced and non-fiber-reinforced direct restorations as long as the biting forces are in a normal range. In the case of extreme forces, direct restorations utilizing flowable SFRC perform better compared to conventional composite fillings. The use of flowable SFRC allows for a favorable fracture profile.

## Figures and Tables

**Table 1:** *p* values of pairwise log-rank post hoc comparisons among tested groups (Kaplan–Meier survival estimator followed by a log-rank test for cycles until failure or the end of the fatigue loading).

Gr	Control		Group 1		Group 2		Group 3		Group 4	
	Chi-Square	Sig.	Chi-Square	Sig.	Chi-Square	Sig.	Chi-Square	Sig.	Chi-Square	Sig.
Control	-	-	0.077	0.781	0.610	0.435	3.387	0.066	0.257	0.612
Group 1	0.077	0.781	-	-	0.183	0.669	1.512	0.219	0.181	0.670
Group 2	0.610	0.435	0.183	0.669	-	-	0.303	0.582	0.006	0.937
Group 3	3.387	0.066	1.512	0.219	0.303	0.582	-	-	0.529	0.467
Group 4	0.257	0.612	0.181	0.670	0.006	0.937	0.529	0.467	-	-

**Table 2:** The distribution of fracture patterns among the tested groups (n = 20).

	Control	Group 1	Group 2	Group 3	Group 4
No fracture	8	9	12	13	12
	40.0%	47.4%	60.0%	65.0%	60.0%
Non-restorable	8	0	0	3	1
	40.0%	0.0%	0.0%	15.0%	5.0%
Restorable	4	11	8	4	7
	20.0%	52.6%	40.0%	20.0%	35.0%

## Data Availability

Data is contained within the article.
